# IL-8 associates with a pro-angiogenic and mesenchymal subtype in glioblastoma

**DOI:** 10.18632/oncotarget.24595

**Published:** 2018-02-28

**Authors:** Siobhan Conroy, Frank A.E. Kruyt, Michiel Wagemakers, Krishna P.L. Bhat, Wilfred F.A. den Dunnen

**Affiliations:** ^1^ Department of Pathology and Medical Biology, Division of Pathology, University of Groningen, University Medical Center Groningen, Groningen, The Netherlands; ^2^ Department of Translational Molecular Pathology, The University of Texas, M.D. Anderson Cancer Center, Houston, TX, USA; ^3^ Department of Neurosurgery, University of Groningen, University Medical Center Groningen, Groningen, The Netherlands; ^4^ Department of Medical Oncology, University of Groningen, University Medical Center Groningen, Groningen, The Netherlands

**Keywords:** glioblastoma, subclasses, angiogenesis, IL-8

## Abstract

Glioblastoma (GBM) is a highly aggressive brain tumor characterized by a high rate of vascularization. However, therapeutic targeting of the vasculature through anti-vascular endothelial growth factor (VEGF) treatment has been disappointing, for which Angiopoietin-2 (Ang-2) upregulation has partly been held accountable. In this study we therefore explored the interplay of Ang-2 and VEGFA and their effect on angiogenesis in GBM, especially in the context of molecular subclasses. In a large patient cohort we identified that especially combined high expression of Ang-2 and VEGFA predicted poor overall survival of GBM patients. The high expression of both factors was also associated with increased IL-8 expression in GBM tissues, but *in vitro* stimulation with Ang-2 and/or VEGFA did not indicate tumor or endothelial cell-specific IL-8 responses. Glioblastoma stem cells (GSCs) of the mesenchymal (MES) subtype showed dramatically higher expression of IL8 when compared to proneural (PN) GSCs. Secreted IL-8 derived from MES GSCs induced endothelial proliferation and tube formation, and the MES GBMs had increased counts of proliferating endothelial cells. Our results highlight a critical pro-angiogenic role of IL-8 in MES GBMs.

## INTRODUCTION

Glioblastoma (GBM) is the most common and aggressive primary brain tumor in adults [1, 2]. Current standard of care comprises maximal safe surgical resection with concurrent chemo-radiation followed by maintenance chemotherapy [3, 4]. Despite application of optimal treatment, the prognosis of patients diagnosed with GBM has remained rather disappointing with a median survival of only 15 months [4]. Development of more effective therapies are therefore urgently needed. Given the highly vascularized nature of GBMs and the correlation between tumor angiogenesis and disease severity [5–7], the potential of therapy targeting the vascular compartment has been intensively studied.

Vascular endothelial growth factor A (VEGFA) is a central player in angiogenesis and can be induced by a variety of angiogenic signaling pathways as well as hypoxia [8–10]. Therapeutic targeting of VEGFA and its corresponding receptors has therefore been assessed broadly. For example, anti-VEGFA therapy using Bevacizumab was initially proven to be successful in recurrent GBM [11, 12], but the results of Phase III Bevacizumab trials in newly diagnosed GBM have provided less encouraging results [13, 14]. Although Bevacizumab treatment in newly diagnosed GBMs resulted in an increase in progression-free survival (PFS), an effect on overall survival (OS) was never observed. This response pattern possibly reflects an initial effect of the therapy, which is later compromised by the development of resistance mechanisms. A potential resistance mechanism could be the exploitation of alternative angiogenic signaling pathways, and due to the strong dependency of VEGF signaling on Angiopoietin/Tie-2 (Ang/Tie-2) signaling, this pathway is of particular interest for treatment resistance.

Ang/Tie-2 signaling involves the competitive binding of the ligands angiopoietin-1 (Ang-1) and angiopoietin-2 (Ang-2) to their receptor Tie-2 [15, 16]. Ang-1 binding to Tie-2 results in vessel stabilization, while Ang-2 binding results either in vessel destabilization in the absence of VEGFA or in vessel proliferation when VEGFA is present. The Ang-1/Ang-2 ratio was found to correlate with OS in GBM patients and high levels of Ang-2 correlated with resistance to anti-VEGFA therapy [17, 18]. Ectopic expression of Ang-2 in GBM cells was found to mediate the diminished effects of anti-VEGF therapy by increasing vascular permeability in mouse models [19]. Alternatively, elevated Ang-2 levels following treatment also increases attraction of bone marrow derived cells that assist perivascularly in the protection of vulnerable vasculature [20]. Therapeutic targeting of Ang-2 has shown broad anti-tumor activity in a number of preclinical solid tumor models, and the addition of anti-Ang-2 treatment to cytotoxic drugs or anti-VEGF treatment has resulted in superior effects over single-agent therapy alone [21, 22]. Two recent studies used this combination approach in preclinical GBM models and both studies indicated superb effects of the addition of Ang2-inhibition to anti-VEGFA monotherapy [23, 24].

In the current study we evaluated the association of Ang-2 and VEGFA expression in GBMs with patient survival and vascularization patterns. The signaling levels through alternate angiogenic signaling pathways were quantified and led to the identification of IL-8 upregulation in pro-angiogenic and MES GBMs. The functional role of this signaling route was explored through *in vitro* angiogenesis assays and immunohistochemistry on patient GBM tissue.

## RESULTS

### High expression of ANGPT2 and VEGFA is associated with a worse prognosis and IL-8 upregulation

**Figure 1:**
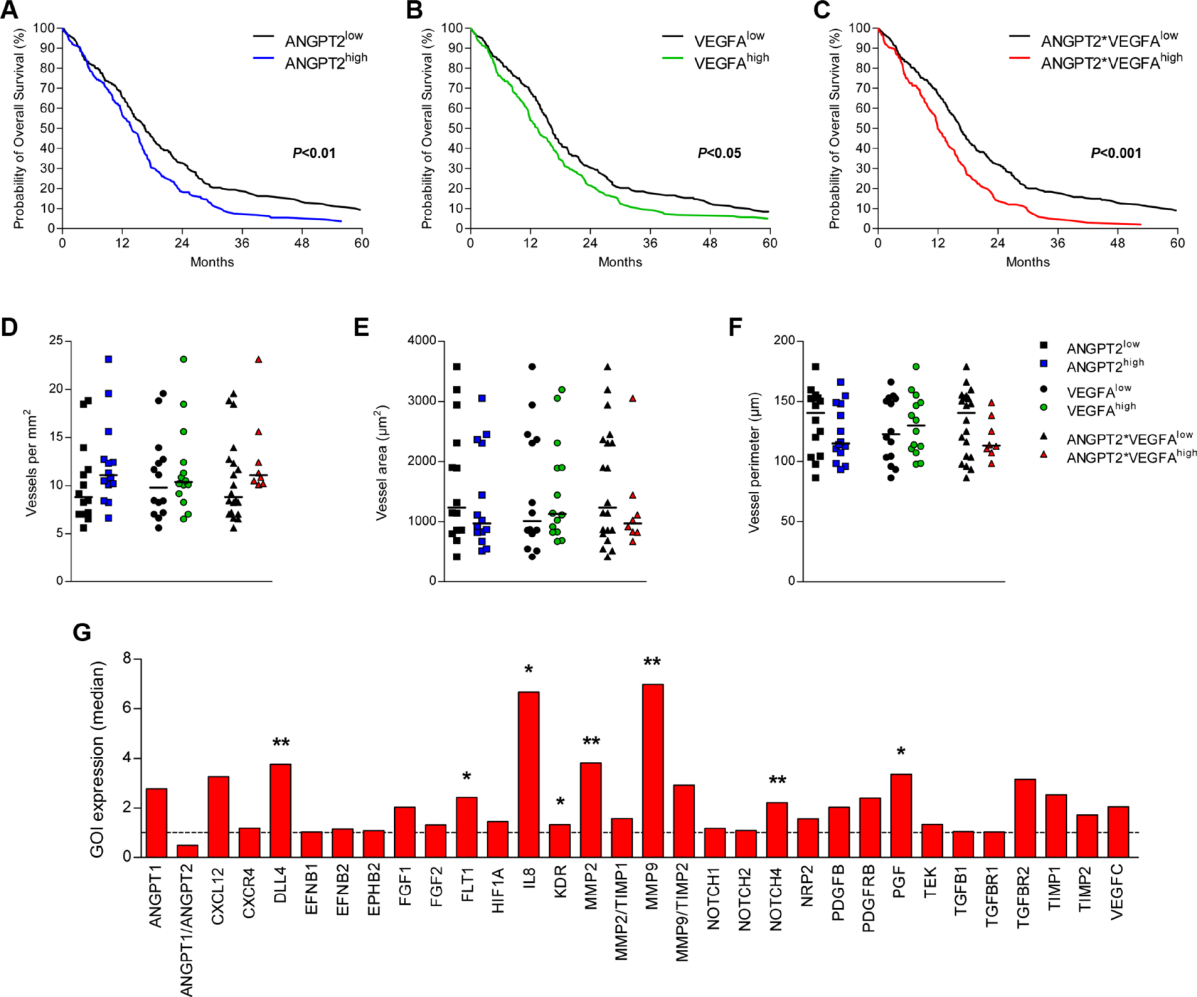
Combined high expression of Ang-2 and VEGFA associates with survival and increased IL-8 expression The expression level of ANGPT2 (**A**), VEGFA (**B**) and the combined high expression of ANGPT2 and VEGFA (**C**) associated with poorer survival of GBM patients (TCGA cohort). The differential expression of these factors (above or below median) did not associate with microvascular density (**D**), vessel size (**E**) or vessel perimeter (**F**) (Groningen cohort). Individual values per patient are displayed and horizontal lines represent median scores. (**G**) Profiling of several angiogenic mRNAs identified IL8 and MMP9 as the targets upregulated most in the tumors with combined high expression of ANGPT2 and VEGFA; ^*^P < 0.05, ^**^P < 0.01.

The expression levels of the pro-angiogenic factors Ang-2 and VEGFA were determined in 525 patients with GBM obtained from the TCGA cohort (*n* = 525) and was examined for possible correlation with OS. Patients were divided into high and low expressing tumors based on the median expression level. The Kaplan-Meier analysis of these groups revealed that high expression of ANGPT2 (*P* < 0.01) and VEGFA (*P* < 0.05) was independently associated with a worse prognosis (Figure 1A, 1B). Then a combination variable was constructed to separate tumors with high expression of both ANGPT2 and VEGFA (ANGPT2*VEGFA^high^) from the other tumors. Kaplan-Meier analysis of this stratification of patients revealed that the combined high expression of these pro-angiogenic factors was also associated with worse survival (*P* < 0.001, Figure 1C).

A Cox regression analysis was performed to compare the prognostic value of ANGPT2, VEGFA and the combined high expression of both factors, which indicated that only the combined high expression maintained significant prognostic value (*P* = 0.040, Table 2). These results were replicated in the REMBRANT cohort (*n* = 151), and subsequently the prognostic value of the angiogenic factors was also assessed in a Cox regression that included the known prognostic factors age at diagnosis and KPS. In this model only age at diagnosis and KPS maintained prognostic significance (Supplementary Table 1). Of the angiogenic factors ANGPT2*VEGFA had the highest HR.

**Table 1:** Summary of GBM patient characteristics in the different cohorts

Characteristic	TCGA cohort	REMBRANDT cohort	Groningen cohort
Number of patients (*n*)	525	151	28
Mean age at diagnosis (95% CI)	58 (56–59)	-	54 (48–59)
Median OS in days (range)	327 (2–3881)	509 (8–3614)	353 (62–1447)
Male sex (%)	320 (61)	79 (62%)^*^	18 (64)
Female sex (%)	205 (39)	48 (38%)^*^	10 (36)

^*^The gender of 24 patients was not available from the database.

**Table 2:** Univariate and multivariate analyses of possible prognostic parameters for OS of GBM patients in the TCGA and REMBRANDT cohort

Characteristic	Univariate analysis		Multivariate analysis	
	HR (95% CI)	*P* value	HR (95% CI)	*P* value
*TCGA cohort (n = 525)*				
ANGPT2	1.344 (1.103–1.637)	**0.003**	1.015 (0.745–1.384)	0.924
VEGFA	1.260 (1.036–1.532)	**0.021**	0.952 (0.712–1.273)	0.740
ANGPT2*VEGFA	1.524 (1.238–1.875)	**<0.001**	1.562 (1.021–2.388)	**0.040**
	
*REMBRANDT cohort (n = 151)*				
ANGPT2	1.212 (0.869–1.690)	**0.256**		
VEGFA	1.261 (0.905–1.758)	**0.171**		
ANGPT2*VEGFA	1.518 (1.055–2.185)	**0.024**		

To further assess the role of Ang-2 and VEGFA in GBM angiogenesis a set of 28 tumors was assembled (Groningen cohort). Similar to the survival analyses, this cohort was also subdivided in groups based on differential expression of ANGPT2, VEGFA or a combination of both. The microvascular density (MVD) was analyzed on tissue sections for the number of vessels per mm^2^, average vessel area (size) and vessel perimeter, but all MVD-parameters did not differ significantly between tumors in association with ANGPT2 and/or VEGFA expression (Figure 1D–1F).

We then continued with profiling of the angiogenic signaling of the Groningen cohort. The comparison of tumors with above median expression of ANGPT2 and VEGFA with the other GBMs showed several significant differences between these groups, with IL8 and MMP9 as the strongest upregulated transcripts in the angiogenic subgroup (ANGPT2*VEGFA^high^, Figure 1G). In addition DLL4, FLT1, MMP2, NOTCH4 and PGF were also found to be upregulated in these tumors. MMP proteins serve an important role in angiogenesis as they degrade the extracellular matrix, of which MMP2 and MMP9 were both significantly upregulated in the ANGPT2*VEGFA^high^ group. As the natural inhibitors of both MMPs were also slightly elevated, the relative increase of both proteinases (MMP2/TIMP1 and MMP9/TIMP2) was not significant. Since IL-8 is well-known for its pro-angiogenic effects we decided to focus on this target.

### The expression of ANGPT2 and VEGFA is associated with IL8 expression

**Figure 2:**
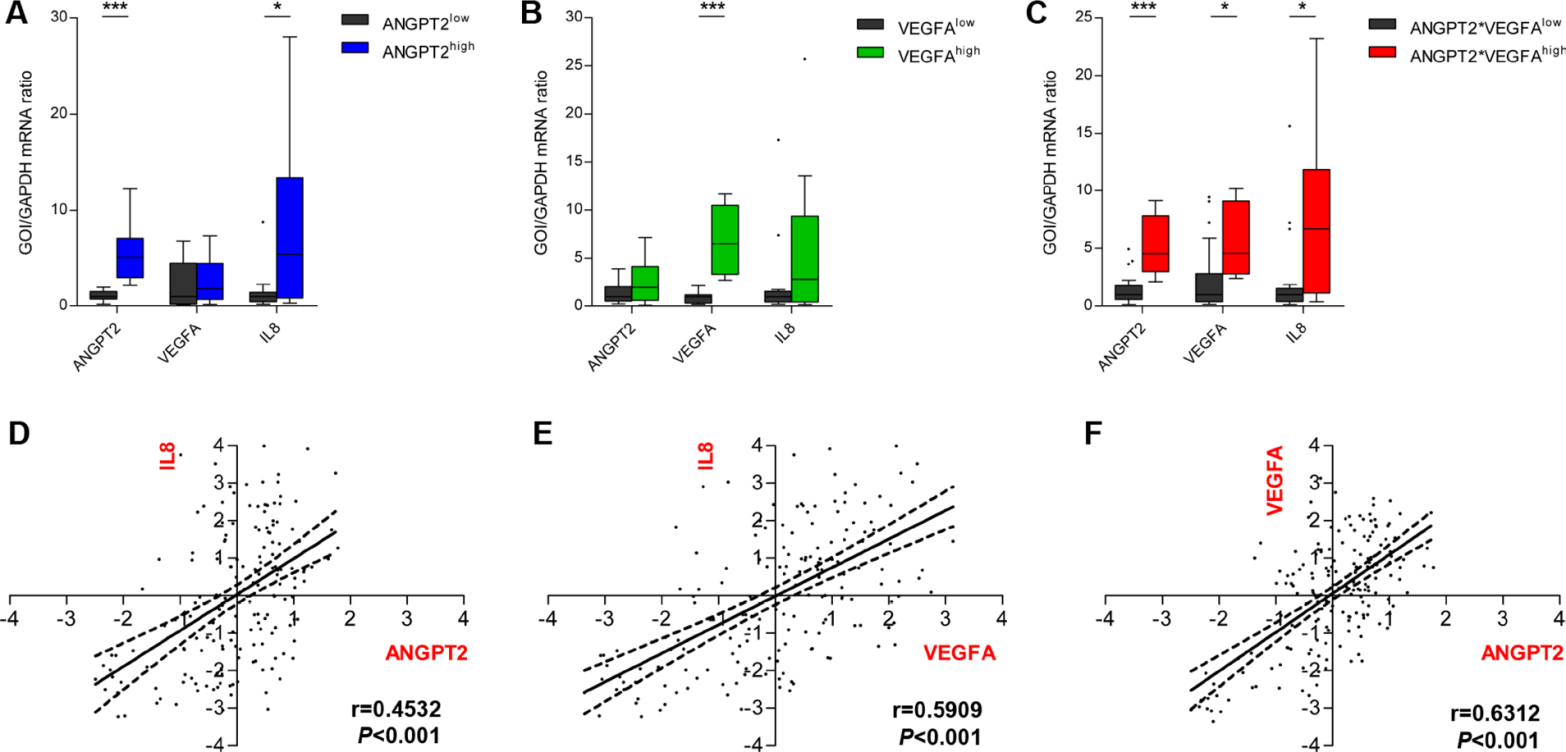
Ang-2 and VEGFA expression associate with increased IL-8 expression In the Groningen cohort tumors with higher than median ANGPT2 expression (**A**) and tumors with higher than median expression of ANGPT2 and VEGFA (**C**) expressed significantly higher levels of IL-8 mRNA as well. A similar but non-significant trend was observed for tumors with above median expression of VEGFA (**B**). Box-and-whiskers were generated according to Tukey’s method with the box representing the 25th and 75th percentile, and whiskers represent 1.5 × IQR. Values outside these intervals are plotted as individual points. The expression levels of ANGPT2 (**D**), VEGFA (**E**) and IL-8 (**F**), explored in the larger TCGA cohort, were all positively correlated; ^*^P < 0.05, ^***^P < 0.001.

The results from the Groningen cohort indicating that IL8 expression is higher in tumors that also have higher than median expression of ANGPT2 and VEGFA was then further explored in both the Groningen and TCGA cohort. In the smaller Groningen cohort, the GBMs were dichotomized as those above or below median ANGPT2 and/or VEGFA expression as described above. In all these groups higher expression levels of ANGPT2 and/or VEGFA were associated with increased IL8 levels (Figure 2A–2C). Similarly, results obtained from the TCGA cohort confirmed the patterns observed in the Groningen cohort (Supplementary Figure 1).

Additionally, correlations between the expression levels of these three factors were assessed in the larger TCGA cohort. Significant positive correlations were identified between ANGPT2 and IL8 (*r* = 0.4532, *P* < 0.001, Figure 2D), VEGFA and IL8 (*r* = 0.5909, *P* < 0.001, Figure 2E) and between ANGPT2 and VEGFA (*r* = 0.6312. *P* < 0.001, Figure 2F). The analyses in the Groningen cohort only reproduced the association between ANGPT2 and IL8 expression (*r* = 0.7093, *P* < 0.01, Online Resource 4), but did not identify any other correlations.

### Effects of Ang-2 and VEGFA stimulation on tumor and endothelial cells

**Figure 3:**
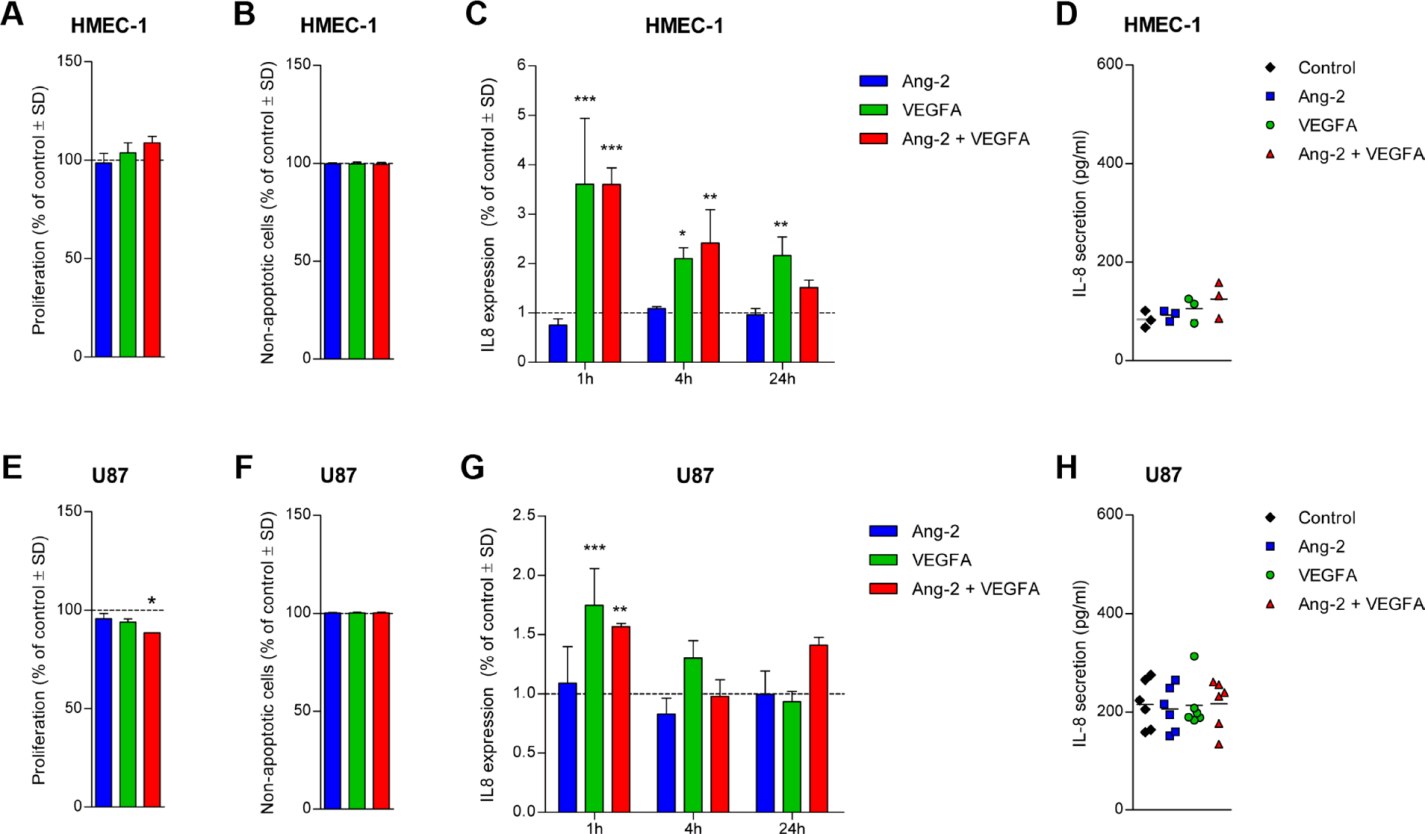
Effect of Ang-2 and/or VEGFA stimulation on U87 GBM cells and HMEC-1 endothelial cells The proliferation (**A**) and rate of CC3-mediated apoptosis (**B**) in HMEC-1 was unaltered following stimulation with Ang-2 and/or VEGFA after 72 and 24 hours, respectively. The transcription of IL8 was upregulated mainly following VEGFA stimulation at different time points (**C**), but the 24-hour secretion level of IL-8 was unaltered in HMEC-1 cells (**D**). The proliferation of U87 cells was slightly inhibited after dual stimulation with Ang-2 and VEGFA (**E**), but CC3-mediated apoptosis levels were unaltered (**F**). VEGFA induced upregulation of IL-8 transcription in U87 only at the 1-hour time point (**G**), but 24-hour secretion levels of IL-8 were indifferent between the conditions (**H**). Assays were repeated three times and mean values ± standard deviations are displayed; ^*^P < 0.05, ^**^P < 0.01, ^***^P < 0.001 all relative to control.

To explore whether the expression level of IL-8 can be modulated by Ang-2 or VEGFA we continued with the stimulation of HMEC-1s using recombinant Ang-2 and VEGFA proteins. The concentration used for Ang-2 (400 ng/ml) and VEGFA (100 ng/ml) were chosen based on results in tube formation assays in which they produced inhibitory and stimulatory effects, respectively (data not shown). The single or dual stimulation did not significantly alter proliferation or apoptosis in HMEC-1 cells (Figure 3A, 3B), but especially VEGFA stimulation induced IL8 transcription 1, 4 and 24 hours after stimulation (Figure 3C). This induction did not translate into significantly different secretion levels of IL-8 protein (Figure 3D).

The dual stimulation of U87 cells inhibited 72-hour proliferation by 11 percent (*P* < 0.05, Figure 3E), but did not induce apoptosis (Figure 3F). The induction of IL8 transcription detected in HMEC-1 was less pronounced in U87 cells treated with Ang-2 and VEGFA (Figure 3G). IL-8 secretion by U87 cells following stimulation was relatively unaffected (Figure 3H), similar to the effects observed in HMEC-1 cells.

### MES subtype associates with increased IL8 expression and IL-8 mediates *in vitro* angiogenesis

**Figure 4:**
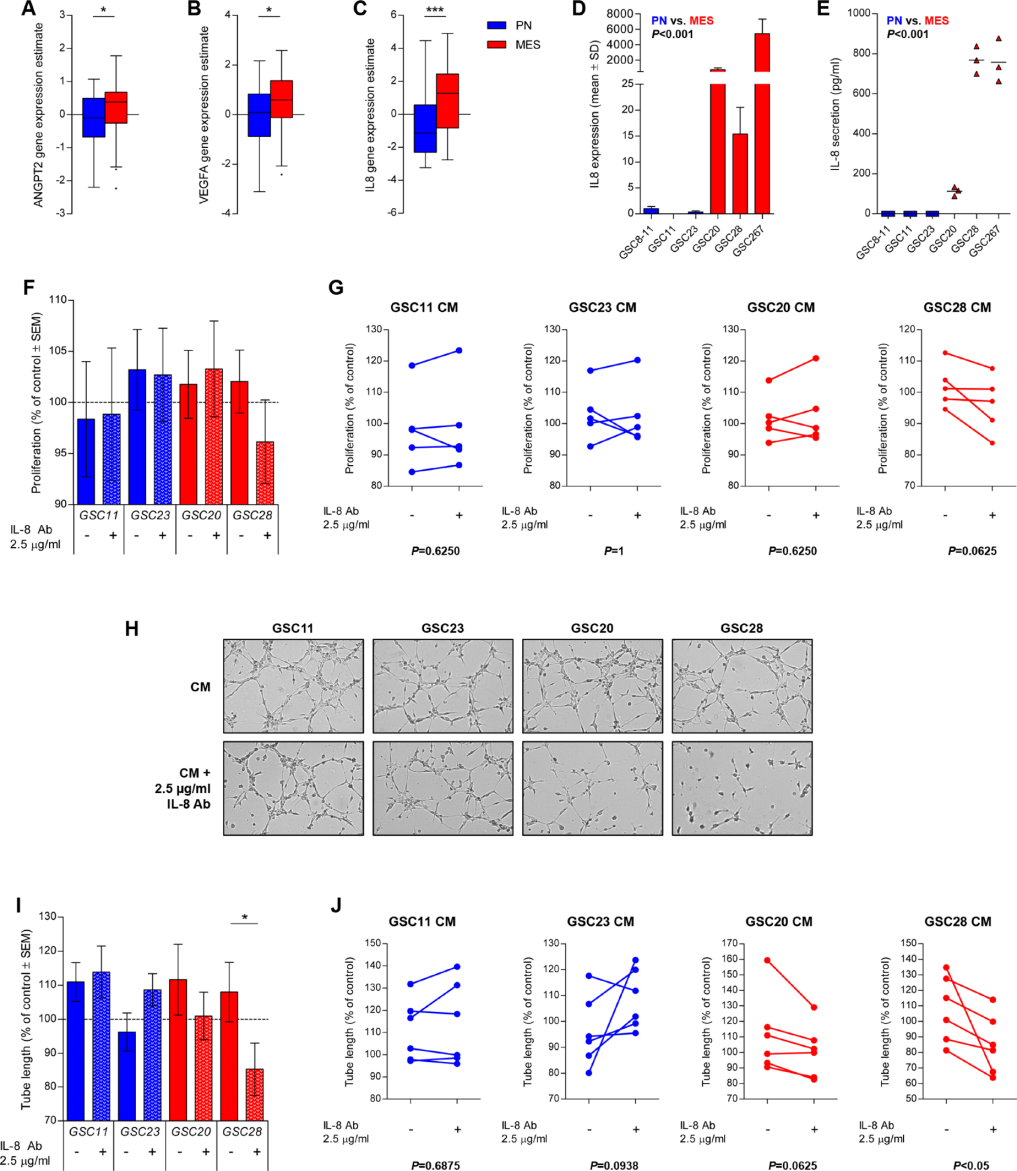
IL-8 is upregulated in MES GSCs and mediates *in vitro* angiogenesis Expression levels of ANGPT2, VEGFA, and IL8 mRNA are upregulated in MES versus PN GBMs (TCGA cohort, **A**–**C**). MES GSCs (GSC 20, 28 and 267) display higher levels of the mRNA transcript in comparison to PN GSCs (GSC 8-11, 11 and 23) (**D**). The secretion level of IL-8 also indicated increased IL-8 secretion by MES GSCs in comparison to PN GSCs (**E**). CM of GSCs only had minor effects on HMEC-1 proliferation, with a trend for inhibition of HMEC-1 proliferation when an IL-8 was neutralized in MES GSC28 CM (**F**). Representative images of tube formation assays are displayed (**G**) that illustrate enhanced tube formation by the addition of MES CM and significant inhibition of tube formation when IL-8 is neutralized in GSC28 CM (**H**). The variation in individual assays is also shown (**I**, **J**). Assays were repeated five (proliferation) or six (tube formation) times and mean values with standard error of the mean (SEM) are plotted; ^*^P < 0.05, ^***^P < 0.001.

We then shifted our focus to GBM stem-like cells (GSCs) that have been shown to better reflect the phenotype of the human disease [25, 26]. GBMs of the MES subtype have been reported to express higher levels of angiogenic markers than their PN counterparts [27]. Analyses of gene expression in MES GBMs showed higher mRNA levels of ANGPT2, VEGFA and IL8 (TCGA cohort, Figure 4A–4C). The TCGA analysis was then followed up with *in vitro* experimentation with a panel of GSCs representative of the PN and MES phenotype that have been described previously [28]. The MES GSCs exhibited a dramatically higher expression of IL8 mRNA in comparison to PN GSCs (*P* < 0.001, Figure 4D). Unlike in HMEC-1s and U87 cells, the mRNA expression in MES GSCs correlated with a concordantly higher IL-8 protein secretion (*P* < 0.001, Figure 4E).

To test whether IL-8 would be capable of inducing a MES transition in PN GSCs we treated PN GSCs (GSC11 and 23) with recombinant human IL-8 and tested the induction of master MES transcriptional regulators at different time points. There were only minor differences between control and stimulated cells regarding STAT3, CEBPB and TAZ expression (Supplementary Figure 2), leading us to conclude that IL-8 did not directly control the MES transition of the PN GSCs. We therefore continued with the exploration of the paracrine effect of PN and MES GSCs on endothelium.

The functional impact of IL-8 secretion on angiogenesis was then further addressed through the use of conditioned medium (CM) from PN and MES GSCs. The treatment of endothelial cells with GSC CM in general did not substantially affect endothelial cell proliferation, but interestingly the addition of an IL-8 neutralizing antibody only resulted in a partial (non-significant) inhibition when added to CM from MES GSC28 (Figure 4F). Similarly, in tube formation assays CM from both GSC subtypes generally enhanced the rate of tube formation compared to controls (Figure 4G, 4H), but the addition of the IL-8 neutralizing antibody only prevented the enhancement of tube formation in the case of MES CM. These results together illustrate that IL-8 is an important mediator of MES-induced angiogenesis in GBM.

### MES GBMs have more proliferating endothelial cells

**Figure 5:**
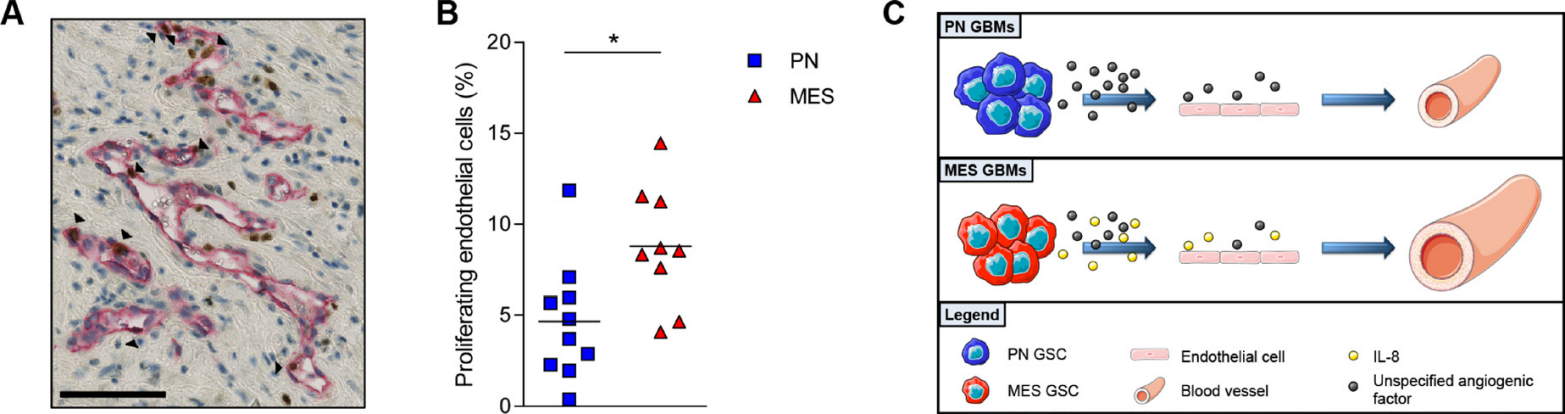
MES GBMs have more proliferating endothelial cells A photomicrograph of a double stained GBM sample for CD34 in red and Ki67 in brown is depicted (**A**). The black arrowheads indicate CD34^+^Ki67^+^ cells. Scale bar is 100 µm. Quantification of the double stained cells indicated that MES GBM tissue had a significantly higher percentage of PECs in comparison to the PN GBM tissues (**B**). (**C**) Proposed model of vessel expansion due to increased IL-8 secretion by MES GSCs ; Scale bar = 100 µm; ^**^P < 0.01.

To test the hypothesis that MES GBMs have higher levels of IL-8 which could serve as signals for angiogenesis, the level of dividing endothelial cells was quantified on GBM tissue from patients. The average number of double-stained cells for CD34 (endothelial marker) and Ki-67 (proliferation marker) was confirmed to be higher in MES GBMs (*P* < 0.01, Figure 5A, 5B). In summary of these findings we propose a model in which MES GSCs secrete IL-8 that affects the survival and proliferation of endothelial cells in a paracrine fashion (Figure 5C).

## DISCUSSION

In previous studies it has been shown that ANGPT2 and VEGFA individually are associated with a worse prognosis for GBM patients [5, 17, 29]. In this study, we showed that the combined higher expression of ANGPT2 and VEGFA is even stronger associated with a worse prognosis in two independent cohorts, although this combination factor did not qualify as an independent prognostic variable in a multivariate analysis including the known prognostic factors age and KPS. Functionally, increased expression of Ang-2 and VEGFA could not be linked to increased vascularity, but the examination of an angiogenic transcriptional profile identified a strong association between high ANGPT2 and VEGFA expression and IL8 expression.

IL-8, a chemokine also known as CXCL8, was initially discovered for its pro-inflammatory functions, but the contributions of IL-8 to other tumorigenic processes are now well documented [30, 31]. IL-8 expression is associated with glioma grade [32], and within GBMs, a 3-fold higher expression of IL-8 was associated with diminished patient survival [33]. The suppression of the upstream regulator ING4 illustrated the pro-tumoral effects of enhanced IL-8-signaling in an *in vivo* GBM model [34], which was tightly related to increased vascularity. Another study confirmed that IL-8 in the secretome of GBM cells was a major pro-angiogenic factor through endothelial CXCR2-signaling [33]. Since we also initially discovered the association between a pro-angiogenic phenotype and elevated IL-8 expression, we focused this study on the role of IL-8 in GBM angiogenesis.

In addition to the association with the pro-angiogenic phenotype, the expression of IL-8 was also found to be elevated in MES GBMs. Molecular GBM subtypes that associate with survival and response to therapy have been described previously, and the MES subtype was initially described as the more vascularized and angiogenic subtype [27, 35]. We have previously reported that MES GBMs do have larger but not necessarily more vessels, and the angiogenic signaling profile of the PN, classical (CLAS) and MES subtypes were rather similar [48]. In this study we looked at the PN and MES subtype and found a clear upregulation of IL8 expression in and secretion from MES GSCs, and we showed that IL-8 is a key mediator of the paracrine pro-angiogenic effect of MES GSCs.

The effects observed with the *in vitro* tube formation however support these conclusions better than the proliferation data. Worth noting in this regard was the requirement of serum addition for HMEC-1 maintenance in proliferation assays but not in tube formation assays, which experimentally complicated the detection of isolated IL-8 effects orchestrated via GSC CM amidst reduced but substantial levels of human and fetal bovine serum in the culture medium during proliferation assays. It is therefore possible that under better controlled conditions the paracrine effect of IL-8 on endothelial proliferation could be stronger, and with the higher secretion levels of IL-8 from MES GSCs it is imaginable that these effects will be more pronounced in GBMs of the MES subclass.

Previous studies in other tumor entities have shed light on the effect of IL-8 on the tumor cell compartment, but since the responses detected in the GSCs were limited we primarily focused on the paracrine effect of IL-8 on endothelial cells. In epithelial cancers, IL-8 has been reported to induce an epithelial-to-mesenchymal transition (EMT) [36], which was also shown in relatively more differentiated GBM models through IL-8-mediated F-actin polymerization [37]. Besides the notion that IL-8 can regulate GSC stemness in the perivascular niche [38], the pro-tumorigenic effects of IL-8 in GBM are quite unclear. Whether GBM cells express the IL-8 receptors (CXCR1 and CXCR2) is an ongoing debate and conflicting data have been reported [30, 39–41]. Since the treatment of our PN GSCs did not induce an upregulation of the important MES transcriptional regulators STAT3, C/EBPβ and TAZ [42, 43], we hypothesize that IL-8 upregulation is not necessarily instructive for the MES subtype of these GSCs, but moreover a side-effect of the subclass transcriptional pattern that is active in the tumor cells. With binding sites present for AP-1 and NF-κB in the promoter region of IL-8, maintenance of IL-8 expression in MES GBMs is likely sustained. GBMs of the MES subtype generally suffer more from hypoxia and in hypoxic glioma cultures, AP-1 functions as a critical oxygen-sensitive transcriptional factor for IL-8 [40, 48], while NF-κB is highly expressed in MES GSCs and regulates the MES differentiation [28].

Another prominent characteristic of MES GBMs is tumor cell invasion of which IL-8 is known to be a potent inducer. An IL-8 neutralizing antibody as well as a CXCR1 blocking antibody significantly inhibited glioma cell invasion *in vitro* [41]. Since it was also shown that IL-8 can directly enhance the protease production in endothelial cells [44], we speculate that targeting of the IL-8 signaling axis could also affect tumor invasiveness.

In summary, we showed that the combined high expression of Ang-2 and VEGFA is a stronger prognostic factor than Ang-2 and VEGFA alone. This pro-angiogenic phenotype is strongly associated with increased IL-8 expression, and we propose that this factor has a key role in pro-angiogenic signaling of tumor cells in MES GBMs. Given the diverse involvement of IL-8 in gliomagenesis, further exploration of its role and potential druggability in especially highly angiogenic and MES GBMs could be a promising path for future studies.

## METHODS

### Patient populations

Data were retrieved of two independent GBM cohorts from public data repositories made available by the Cancer Research Network (TCGA, http://cancergenome.nih.gov/) and the National Cancer Institute of the United States (Repository for Molecular Brain Neoplasia Data [REMBRANDT]) [45, 46]. The third cohort concerned the Groningen cohort that comprised an additional set of 28 GBMs on which the vascular assessments were performed. The details and clinical characteristics of the cohorts are summarized in Table 1, and additional experimental details are reported in the Supplementary Materials.

All experiments including the use of human tissue were in accord with the Declaration of Helsinki and were conducted under the ‘code of conduct for dealing responsibly with human tissue in the context of health research’ published by the Federation of Dutch Medical Scientific Societies in 2011 [47]. The research was performed on the leftover material and all tissues were handled coded and anonymously.

### Survival analyses

For the survival analyses the TCGA and REMBRANDT cohort were stratified into below and above median expression groups for Ang-2 and VEGFA expression. The combined high expression group (both Ang-2 and VEGFA above median) was compared against tumors with either one or both Ang-2 and VEGFA expressed below median. The significance of Ang-2 and/or VEGFA expression for survival was assessed by log-rank testing (univariate) and Cox regressions (multivariate), and compared with the known prognostic factors age at diagnosis (≤ or >55 years) and Karnofsky performance scores (KPS, ≤ or >60). All variables with a significance level ≤0.05 were included in the multivariate analysis.

### Immunohistochemistry

GBM tissue sections were stained with antibodies against α-SMA (1A4, Dako, Glostrup, Denmark) and ColIV (MP Biomedicals, Santa Ana, CA, USA). The staining for α-SMA and ColIV were analyzed as described previously for the assessment of vascular parameters [48]. For the quantification of proliferating endothelial cells tissue sections from proneural (PN) and mesenchymal (MES) GBMs were double-stained for CD34 (QBEnd10, Beckman Coulter, Marseille, France) and Ki-67 (MIB1, Dako).

### qRT-PCR

RNA was purified from snap-frozen GBM samples or *in vitro* tissue cultures and reverse-transcribed to cDNA. Samples were analyzed either using previously described custom-designed Taqman Micro Fluidic Cards (Applied Biosystems, Foster City, CA, USA) [48] or individual gene expression (Taqman) assays for IL8, STAT3, CEBPB, TAZ, RPS27 or GAPDH on a Viia^™^ 7 real-time PCR system (Applied Biosystems).

### Cell culture and recombinant proteins

U87 cells were maintained in DMEM/F-12 (Lonza, Verviers, Belgium) supplemented with 1% fetal bovine serum (FBS, Sigma-Aldrich, Munich, Germany) and 1% penicillin/streptomycin solution (pen/strep, Lonza). Human dermal microvascular endothelial cells (HMEC-1) were kindly provided by Dr. E.W. Ades (CDC, Atlanta, GA, USA) [49] via Prof. G. Molema and the UMCG Endothelial Cell Facility and maintained in M-199 medium (Lonza) supplemented with 10% FBS, 10% human serum (Sigma-Aldrich), pen/strep and L-glutamine (Lonza). GSCs were maintained in DMEM/F12 (Lonza) supplemented with 10% B27 (Life Technologies, Bleiswijk, the Netherlands), 20 ng/ml bFGF and EGF (Life Technologies) and pen/strep. Carrier-free recombinant human VEGFA_165_ (from here on referred to as VEGFA), Ang-2 and IL-8 were obtained commercially (R&D Systems, Minneapolis, MN, USA).

### Proliferation assay

For the evaluation of effects on proliferation the Chemicon^®^ 3-(4,5-dimethylthiazol-2-yl)-2,5-diphenyl tetrasodium bromide (MTT) cell proliferation assay was performed according to the manufacturer’s instructions (Merck Millipore, Darmstadt, Gemany). Each condition was assessed in triplicate and averaged data from three independent assays are plotted relative to the control.

### Apoptosis assay

Effects on apoptosis were assessed through staining for Cleaved Caspase 3 (Clone Asp175, Cell Signaling Technology, Danvers, MA, USA). A total of 5 images was quantified per condition and averaged data from three independent experiments are plotted relative to the control.

### Protein secretion

IL-8 protein secretion levels were measured using a human IL-8 enzyme-linked immunosorbent assay (ELISA) according to the manufacturer’s protocol (R&D Systems).

### Capillary-like tube formation assay

After 24 hour serum starvation 5 × 10^3^ HMEC-1 cells were seeded on growth factor-reduced Matrigel^®^ in µ-Slides for Angiogenesis (Ibidi, Munich, Germany). Recombinant proteins (Ang-2 and/or VEGFA) were added to serum-free HMEC-1 medium at indicated amounts, or in the case of CM experiments the GSC CM was added to HMEC-1 cells cultured in serum-free medium with a minimal CM concentration of 75%. After 5 hours incubation at 37° C each well was photographed and image analysis was carried out using Angiogenesis Analyzer [50] in ImageJ.

### Statistical analysis

All statistical analyses were performed using SPSS software version 22.0 (SPSS, Chicago, IL, USA), and visualized using Graphpad Prism version 5 (Graphpad Software Inc, San Diego, CA, USA). Differences between groups were determined by a one-way ANOVA or *t*-test when data were normally distributed, and by a Kruskal–Wallis test or Mann–Whitney *U* test when data were not normally distributed. The multiple group comparisons were followed up by either Tukey’s or Dunn’s post-hoc tests. Reported correlations concern Pearson r correlations. *P*-values < 0.05 were considered significant and in all cases exact two-sided *P*-values were reported.

## SUPPLEMENTARY MATERIALS FIGURES AND TABLE


