# Supplementary material for: IL-8 associates with a pro-angiogenic and mesenchymal subtype in glioblastoma
Source: Oncotarget. 2018 Feb 28;9(21):15721–31. doi: 10.18632/oncotarget.24595 (PMC5884659; doi:10.18632/oncotarget.24595)
Supplement: Supplementary file 1 [file oncotarget-09-15721-s001.pdf]

# IL-8 associates with a pro-angiogenic and mesenchymal subtype in glioblastoma

## SUPPLEMENTARY MATERIALS

### Patient populations and survival analyses

For both the TCGA cohort and the REMBRANDT cohort gene expression data obtained with the Genechip Human Genome HT-HG-U133A platform were retrieved. For the TCGA cohort this concerned a total of 525 patients from who level 3 gene expression data and clinical characteristics including survival. We defined survival as the time interval from surgery until the date of death. Since the KPS value was missing for 139 of the 525 patients, the analysis reported in Supplementary Table 1 was performed on the remaining 386 patients of the TCGA tumor set.

The gene expression data for the REMBRANDT cohort with accompanying clinical characteristics were available for 151 GBM patients, and the analysis of the data from this database was performed using BRB-ArrayTools developed by Dr. Richard Simon and BRB-ArrayTools Development Team. Log-transformation and median centering of the genes was performed. Because age at diagnosis was only available as a range parameter of 5 years this variable was excluded from further analyses, while the gender status was missing for 24 patients.

All patients in the Groningen cohort underwent surgical debulking in the University Medical Center Groningen in the period August 2006 to May 2012.

### CM preparation

GBM cells were stimulated with recombinant human Ang-2 and/or VEGFA for 6 hours, after which the cells were washed with Hank's Balanced Salt Solution (HBSS, Lonza) and fresh serum-free culture medium was added. Supernatant were collected after 24 hours of conditioning and filtered through 0.22 µm filters (Corning, New York, NY, USA) and applied as indicated in functional *in vitro* experimentation.

### Proliferation assay

For the proliferation assays $2.5 \times 10^3$ cells were seeded in 96-wells plates and treated as indicated. MTT reagent was added after 72 hours, incubated with the cells for 4 hours and formazan crystals were formed. Then 0.04N HCl in isopropanol was added and thoroughly mixed to dissolve the formazan. Directly after dissolving the absorbance was measured at 570 nm and a reference wavelength of 630 nm (Varioskan, Thermo Fisher Scientific, Waltham, MA, USA).

For the proliferation assays using GSC CM M199 medium was mixed with GSC CM in equal amounts, and human serum and fetal bovine serum were added to these mixtures in a final concentration of 1.25%.

### Apoptosis assay

To assess the induction of apoptosis following stimulation cells were seeded in chamber slides (Thermo Fisher Scientific) and stimulated with recombinant proteins or conditioned medium for 24 hours. Then the cells were fixed using 4% paraformaldehyde and stained for Cleaved Caspase-3 through immunofluorescent staining. Aspecific binding of antibodies was blocked using 0.2% non-fat dry milk in phosphate-buffered saline (PBS) for 30 minutes. Then slides were incubated with 1:400 diluted rabbit anti-Cleaved Caspase-3 antibody (Clone Asp175, Cell Signaling Technology, Danvers, MA, USA). Cells were washed with PBS and incubated with a 1:200 diluted goat anti-rabbit antibody with an Alexa Fluor 555 label (Life Technologies, Carlsbad, CA, USA) for 30 minutes. After another PBS wash nuclei were counterstained with DAPI. The experiment was repeated three times and in each experiment A172 GBM cells treated with recombinant human sTRAIL (100 ng/ml for 24 hours, Preprotech, Rocky Hill, NJ, USA) were used as a positive control. A total of 5 images at 10× magnification were obtained per condition, from which the Cleaved Caspase-3-negative fraction was quantified.

**Supplementary Table 1: Univariate and multivariate analyses of possible and known prognostic parameters for OS of GBM patients in the TCGA cohort**

| Characteristic | Univariate analysis | | Multivariate analysis | |
|---|---|---|---|---|
| | **HR (95% CI)** | ***P* value** | **HR (95% CI)** | ***P* value** |
| *TCGA cohort (n = 386)* | | | | |
| KPS | 2.330 (1.778–3.052) | **<0.001** | 1.978 (1.497–2.613) | **<0.001** |
| Age at diagnosis | 2.086 (1.632–2.666) | **<0.001** | 1.777 (1.374–2.297) | **<0.001** |
| ANGPT2 | 1.394 (1.103–1.762) | **0.005** | 1.024 (0.703–1.491) | 0.903 |
| VEGFA | 1.335 (1.060–1.683) | **0.014** | 0.883 (0.634–1.231) | 0.464 |
| ANGPT2*VEGFA | 1.645 (1.282–2.110) | **<0.001** | 1.445 (0.870–2.399) | 0.155 |

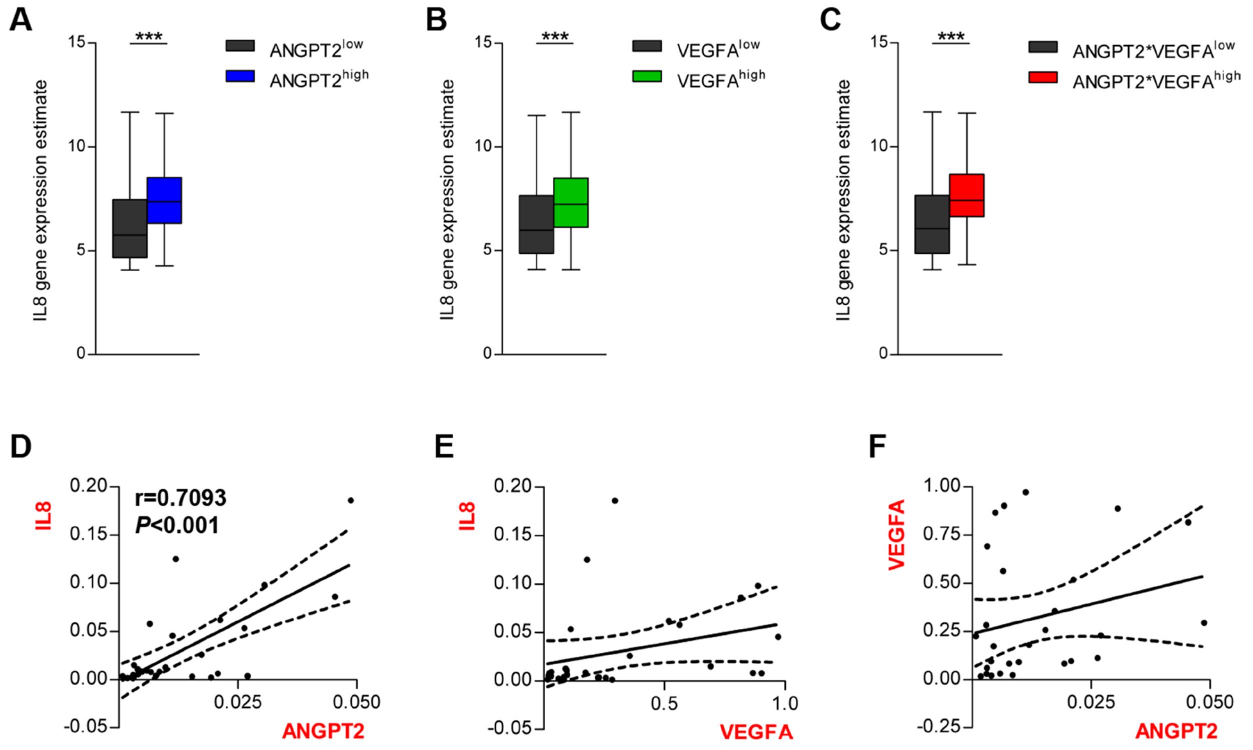


**Supplementary Figure 1: Expression of ANGPT2 and VEGFA associates with increased IL8 expression in the TCGA cohort.** In the TCGA cohort tumors with higher than median expression of ANGPT2 (**A**), VEGFA (**B**) or both ANGPT2 and VEGFA (**C**) all expressed significantly higher levels of IL-8 mRNA. The correlation between ANGPT2 and IL8 was significant ($r = 0.7093$, $P < 0.001$) (**D**), while VEGFA and IL8 as well as ANGPT2 and IL8 were not significantly associated (**E**, **F**).

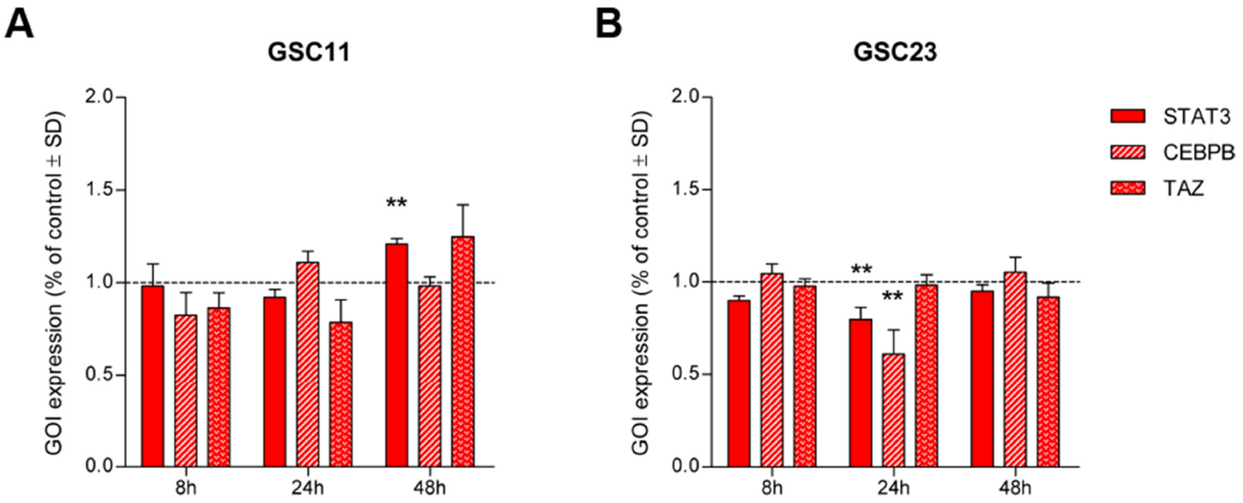


**Supplementary Figure 2: MES transcriptional regulators are not induced in PN GSCs following IL-8 stimulation.** The stimulation of GSC11 (**A**) and GSC23 (**B**) with 100 ng/ml IL-8 did not induce strong upregulation of master MES transcription factors after 8, 24 or 48 hours.
